# When Older Males Sire More Offspring—Increased Attractiveness or Higher Fertility?

**DOI:** 10.1007/s00265-022-03170-0

**Published:** 2022-04-23

**Authors:** Jan T. Lifjeld, Oddmund Kleven, Frode Fossøy, Frode Jacobsen, Terje Laskemoen, Geir Rudolfsen, Raleigh J. Robertson

**Affiliations:** 1 Natural History Museum, University of Oslo, Norway; 2 Norwegian Institute for Nature Research, Trondheim, Norway; 3 Agency for Urban Development, City of Oslo, Norway; 4 Department of Arctic and Marine Biology, The Arctic University of Norway, Tromsø, Norway; 5 The Arctic University Museum of Norway, The Arctic University of Norway, Tromsø, Norway; 6 Department of Biology, Queen’s University, Kingston, ON Canada

**Keywords:** Extrapair paternity, Life history, Sexual selection, Sperm traits, Tail length, Testes size

## Abstract

**Abstract:**

In birds with extrapair mating, older males usually have higher fertilization success than younger males. Two hypotheses can potentially explain this pattern: 1) females prefer older, and often more ornamented males, or 2) older males invest more in reproduction and fertility than younger males. Here we studied factors associated with age-related male fertilization success in a population of barn swallows *Hirundo rustica* in Canada. We document that male fertilization success increased gradually up to a minimum age of four-year old. The age effect was especially strong for the number of extrapair offspring obtained and the occurrence of a second brood. The higher fertilization success of older males was also associated with an early start of breeding in spring. The length of the elongated outermost tail feathers, a postulated male ornament preferred by females, also increased with age (in both sexes), but it was not a significant predictor of male fertilization success within age classes. Male fertility traits, especially testis size, but also sperm motility and sperm velocity, increased significantly across age groups. Our results suggest that the higher fertilization success by older males is due to their higher reproductive investments and that their longer tails are an adaptation to early arrival on the breeding grounds.

**Significance statement:**

The barn swallow is a socially monogamous passerine with extensive extrapair mating. We found that males become more successful in siring both withinpair and extrapair offspring as they become older. Their increased fertilization success was associated with a higher reproductive effort as indicated by larger testes, more motile sperm, and an earlier start of breeding in spring. The length of the outer tail feathers increased with age in both sexes, but long tails did not enhance male fertilization success among males of the same age. Long tails are probably an adaptation to rapid migration and earlier arrival on the breeding grounds. Our findings suggest that the commonly observed age-related increase in male fertilization success in passerine birds is better explained by life history theory than by sexual selection theory.

**Supplementary Information:**

The online version contains supplementary material available at 10.1007/s00265-022-03170-0.

## Introduction

For more than three decades, extrapair paternity has been studied and characterized in hundreds of species of socially monogamous birds, but the ecology and evolution of this mating system is still poorly understood (Griffith et al. 2002; Brouwer and Griffith 2019; Lifjeld et al. 2019; Valcu et al. 2021). A possible reason for the lack of progress is that it is not yet clear whether the behaviour is solely driven by the obvious fitness benefits to males of siring more offspring, or whether female promiscuity is adaptive through enhanced genetic quality of extrapair offspring (Arnqvist and Kirkpatrick 2005; Griffith 2007; Forstmeier et al. 2014). Irrespective of which sex is driving the behaviour, there is a general understanding that whenever certain males are more successful in securing withinpair paternity and achieving extrapair paternity, the variance in male fertilization success will increase the opportunity for sexual selection on the male traits involved (Webster et al. 1995; Whittingham and Dunn 2005). Many paternity studies in birds have therefore searched for associations between fertilization success and male sexual traits (e.g. ornaments), but the overall empirical support for such relationships is rather weak (Griffith et al. 2002; Hsu et al. 2015). A more common predictor of male fertilization success is male age; older males are generally more successful in obtaining extrapair paternity, albeit not necessarily better at defending their withinpair paternity (reviews: Akcay and Roughgarden 2007; Cleasby and Nakagawa 2012; Hsu et al. 2015). This raises the question of how older males achieve higher fertilization success. Are they more attractive to females or are they better at competing for fertilizations, for example, by being more fertile?

Females may have an adaptive preference for older males as sires for their offspring. A possible reason is that older individuals have proven their ability to survive and may therefore transfer better viability genes to offspring (Kokko 1998; Brooks and Kemp 2001). Exaggerated sexual traits (i.e. ornaments) can be a cue for females to target older males, given that the traits show age-dependent expression. We name this the “sexual selection” hypothesis. It predicts that the trait should be more strongly correlated with male fertilization success than age itself and show positive correlation with fertilization success also within male age groups with among-male variation in tail length. The correlation should be particularly strong within the younger age groups, since selection has not yet filtered out the poorer-quality males. Alternatively, older males have higher fertilization success because they invest more time and resources in getting access to mates and competing for fertilizations. They could also perform better, i.e. higher pay-off per investment, due to previous breeding experience (Weatherhead and Boag 1995). This hypothesis can therefore predict a shift in male time budgets and fertility traits with age. Some studies have shown that older males spend more effort on mate attraction and less on mate guarding (Johnsen et al. 2003; Kleven et al. 2006a). Older males have also bigger testes and produce more sperm and larger ejaculates than younger males (Birkhead et al. 1997; Graves 2004; Laskemoen et al. 2008). Girndt et al. (2019) found that old house sparrow *Passer domesticus* males delivered almost three times more sperm to the female’s egg than young males. Higher sperm production gives a numerical advantage in sperm competition (the raffle principle) and, hence, possibly increased fertilization success (Parker 1993). In a previous study on male tree swallows *Tachycineta bicolor*, a species with high levels of sperm competition, males with a large cloacal protuberance (a proxy for higher sperm production), and sperm with a relatively long midpiece had increased fertilizations success (Laskemoen et al. 2010).

Explanations for male fertilization success that focus on male fertility factors have been termed the “male manipulation” hypothesis (Hsu et al. 2015) or the “sugar-free daddy” hypothesis (Nakagawa et al. 2015). We prefer, however, the “life history” hypothesis (sensu Lifjeld et al. 2011), because it emphasizes the within-male shift in reproductive effort with age. Classical life history theory (Williams 1966; Stearns 1992) holds that natural selection will favour the optimization of the major life history traits (survival, growth, and reproduction) over the life span of iteroparous organisms. Individuals should withhold reproductive effort early in life when there is a significant cost to reproduction and the prospects for future reproduction are high (Charlesworth and Leon 1976). The “life history” hypothesis predicts that older males should have higher sperm production (e.g. larger testes) and/or higher sperm quality (e.g. faster or more motile sperm). Note that the hypothesis is indifferent to any female preferences and considers female gametes only as targets for competition among males. Nevertheless, it does not preclude the existence of any female preference on other individual male traits not related to age.

Here we study how male fertilization success varies with age in a population of barn swallows *Hirundo rustica* in Canada. We especially examine how a postulated sexually selected ornament, i.e. the length of the outermost tail feathers (Smith and Montgomerie 1991; Smith et al. 1991), and various fertility measures covary with fertilization success within and among male age groups. In this population, about one-third of all offspring are sired by extrapair males and many pairs are double-brooded (Lifjeld et al. 2011). We have monitored paternity in this population over four consecutive years (2003 – 2006); the results from the first two years have been published previously. In the first year (2003), when male age was unknown, we documented that males with longer tails had higher extrapair fertilization success (Kleven et al. 2006b). In the second year (2004), we found that males returning from the previous breeding season (> 1y-olds) had higher fertilization success than newly established breeders (= 1y-olds). Returning males also had longer tails, more rufous-coloured underparts, and started breeding earlier (Lifjeld et al. 2011). Plumage colour was not a significant predictor of male fertilization success, nor was tail length within the group of 1y-olds. However, tail length correlated positively with fertilization success in the group of older males. Since this group presumably encompassed several age classes, we were not able to determine whether tail length predicted fertilization success among males of the same age (Lifjeld et al. 2011). Hence, the results from the first two years suggested that age was a strong predictor of male fertilization success, but we could not examine any age effects among males older than one year.

The present study analyses patterns of male fertilization success in the following two years when males could be divided into three (2005) or four (2006) age groups according to which year they were first ringed as a breeder. We were then able to examine age-related male fertilization success in more detail, and test whether male tail length was a significant predictor of fertilization success within age classes as predicted by the “sexual selection” hypothesis. Furthermore, in 2006, we obtained data on several male fertility traits: testes size, sperm size, sperm swimming speed, and sperm motility. We use these data to test whether males show age-related increase in fertility as predicted by the “life history” hypothesis.

## Methods

We studied barn swallows at four breeding colonies in the vicinity of Queen’s University Biological Station in Ontario, Canada, during the 2005 and 2006 breeding seasons. Previous papers give details about the study area and the field methods for catching, marking, measuring, and sampling adults and their offspring (Kleven et al. 2005, 2006b, c), and the protocols for paternity analyses (Kleven et al. 2005), sperm length measurements, and motility analyses (Laskemoen et al. 2013a, b). We therefore briefly outline these general methods here. We also note that it was not possible to record data blind because our study involved focal animals in the field.

Adults were caught in mist nets in their breeding colony, ringed, and given a unique colour code of acrylic paint on the front of the right wing for field identification at their nest. Sex was determined based on the shape of the cloacal protuberance, tail measurements, and observations of behaviour at the nest, but sex was also confirmed molecularly (Kleven et al. 2006b). Barn swallows show very high breeding site tenacity (Møller 1994), and about 50% of the breeders return the next year (Kleven et al. 2005). Since we caught almost all breeders each year, we assume that unringed birds were 1y-olds and that previously ringed birds could be aged from their year of ringing. We measured several body and plumage characters, but here we report on wing length and tail length. Wing length was measured as straightened and flattened chord to the nearest 0.5 mm. Tail streamers were measured on both sides to the nearest 0.5 mm and the mean value was used. If one of the feathers was broken or missing (9% of females, 8% of males), we used the value for the intact side. All morphological measurements were taken by the same person (OK).

Ejaculates were collected by cloacal massage from males in 2006 only. Samples were diluted in pre-heated buffer and videofilmed at 40 °C in a microscope in the field. The videos were subsequently analysed with the HTM-CEROS II Sperm Analyzer software (Hamilton Thorne Research, Beverly). Sperm swimming speed was expressed as the curvilinear velocity (VCL) and sperm motility as the proportion of motile cells. An aliquot of the ejaculate sample was preserved in 5% formaldehyde for subsequent sperm length measurements. For each of 10 sperm cells, we measured the length of the head, the midpiece and the posterior part of the flagellum extending from the end of the midpiece, and calculated total sperm length as the sum of the three measurements. Mean values based on the 10 cells were used for each male. Measuring 10 spermatozoa has been shown to give an adequate estimate of a sample’s mean total sperm length (Laskemoen et al. 2007).

A small blood sample was taken from all adults and nestlings, and DNA extracted for parentage analysis. We also extracted DNA from tissues of dead nestlings or unhatched eggs. Paternity was assigned by a panel of six microsatellite loci (Aar4, Hansson et al. 2000; Escµ6, Hanotte et al. 1994; HrU5, HrU6 and HrU7, Primmer et al. 1995; HrU10, Primmer et al. 1996) following the protocol described in Kleven et al. (2005). In a single case, a nestling had two possible sires, a father or his son, that could not be distinguished using the standard microsatellite panel. We genotyped the offspring and the candidate parents at an additional microsatellite locus (Titgata02, Wang et al. 2005) to successfully identify the true sire.

In 2005, breeding colonies were monitored during the entire breeding season (May–August) which encompassed the laying of second clutches for many pairs. The dataset on male fertilization success included 64 male breeders whose broods were paternity tested (90 broods altogether). In addition, 38 males were caught, but did not have any paternity-tested clutch. They were included in the trait analyses, but not in the analyses of fertilization success. In 2006, only first clutches (May–June) were monitored and fertilization success measured for a total of 55 males. Two early second broods were also paternity tested; the extrapair young (EPY) in these broods were included in the estimates of male extrapair paternity success, but withinpair young (WPY) were excluded (i.e. all 55 males had only one own clutch each). Another 46 males were caught that year, but without a paternity-tested first brood. They were included in the analyses of age-related traits, but not in the analyses of fertilization success. A total of 86 males were successfully analysed for sperm morphology and 63 males for sperm velocity and motility.

On 28^th^ and 29^th^ June 2006, 24 males, six from each age group, were collected for analysis of testis size variation. The left and right testes were weighed separately to the nearest 0.01 g and the combined mass used as the measure of testes size.

All statistical analyses and graphical presentations were performed in R (< www.r-project.org >) version 4.0.2. For analyses of age-related changes, we scored age classes as ordinal numerals and used linear models. In multivariate models, we removed non-significant interaction effects. In two-group t-tests, we tested for unequal variances and used the Welch test whenever the two groups had significantly different variances.

## Results

### Male age and fertilization success

The frequency of EPY was similar in the two study years, i.e. 25% (101 EPY among 397 young, N = 90 broods) in 2005 and 23% (62 EPY among 268 young, N = 57 broods) in 2006. The percentage of EPY was nearly identical for first (25%) and second broods (26%) in 2005. Overall, we were able to identify the sire of 94% (153/163) of the EPY.

**Fig. 1:**
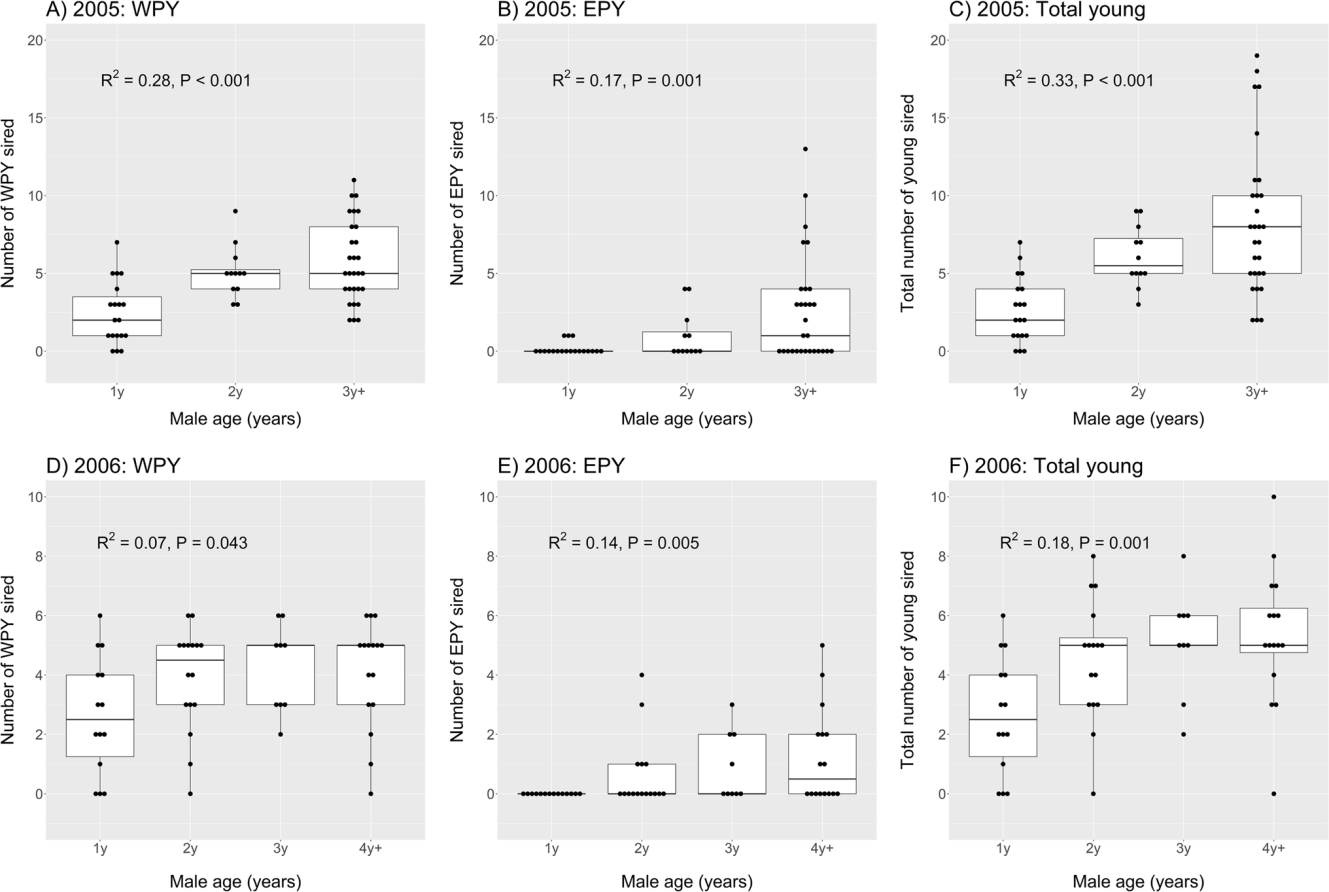
Box plots of age-related fertilization success in male barn swallows in each of two study years (2005 and 2006). Fertilization success is expressed as the number of withinpair young (WPY) sired (panels A and D), the number extrapair young (EPY) sired (panels B and E), and the total number of young (WPY + EPY) sired (panels C and F). Male age is defined from the year of first breeding (e.g. 1y = 1 year old, 4y +  = 4 years, or older). Boxes indicate the interquartile range (IQR), with the central line depicting the median and the whiskers extending to 1.5*IQR, and outliers. The results of linear regression models are indicated, where R^2^ expresses the proportion of variance in male fertilization success explained by male age (scored as ordinal numbers)

**Fig. 2:**
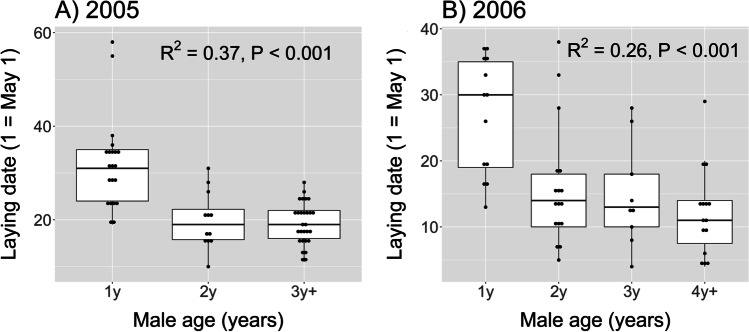
Box plots of the start of egg laying in barn swallows in relation to male age in the two study years (2005 and 2006). Laying date denotes the laying of the first egg. Male age is defined from the year of first breeding (e.g. 1y = 1 year old, 4y +  = 4 years, or older). Boxes indicate the interquartile range (IQR), with the central line depicting the median and the whiskers extending to 1.5*IQR, and outliers. The results of linear regression models are indicated, where R^2^ expresses the proportion of variance in laying date explained by male age (scored as ordinal numbers)

Male total fertilization success increased with age in both field seasons, and the age effect was significant for both the number of WPY and the number of EPY (Fig. 1, Table 2). For the number of WPY, the age effect was stronger in 2005 than in 2006 (cf. Figure 1A versus Fig. 1D). This was due to a higher proportion of older males having two broods in 2005: 62% (18/29) of 3y + males, 33% (4/12) of 2y males, and 11% (2/19) of 1y males (Chi-square = 12.98, P = 0.002) produced a second brood. In 2006, when the WPY number only refers to first broods, the age effect was marginally significant (P = 0.043; Fig. 1D) and caused mainly by the group of 1y males having few WPY. The age-related pattern in the number of WPY was paralleled by an age-related pattern in brood size (F_1,53_ = 4.87, P = 0.032). That is, older males had larger broods, since there was a seasonal decline in brood size and older males started breeding earlier. In both years, older males had an earlier start of egg laying (Fig. 2). In 2005, the probability of having a second brood was associated with both male age and the start of egg laying. An early start of egg laying is probably the more important factor of the two, affecting the likelihood of having a second brood. In a logistic regression of whether a second brood was initiated, and with both male age and egg-laying date of the first clutch as predictors, egg-laying date was significant (z = -3.16, P = 0.002), whereas male age was not (z = 1.09, P = 0.28). Hence, older males seem to sire more WPY because they start breeding early and pair with females that lay larger first clutches and have a stronger tendency to produce a second clutch.

Older males also had a higher success in obtaining fertilizations in other males’ nests. The number of EPY sired increased with male age in both years, and nearly none of 1y males had any EPY (Fig. 1B, E). If we ignore the first-time breeders, 3y + males had significantly more EPY than 2y males in 2005 (Welch t-test: t_38.6_ = 2.08, P = 0.044), but not significantly so in 2006 (t_39_ = 1.13, P = 0.26; the group of 3y and 4y + combined). However, the latter test only includes males with a paternity score for their own brood. If we include all males in the comparison, the difference in EPY number between 3y + and 2y males increased but was still not statistically significant (t_50.6_ = 1.87, P = 0.068). There was no difference in EPY number between 4y + and 3y males (t_28_ = 1.07, P = 0.28).

The total fertilization success (WPY + EPY) therefore also increased with male age in both years (Fig. 1C, F). The increment in total fertilization success was largest between 1 and 2y males in both years. The increment between 2 and 3y + males was also significant in 2005 (Welch t-test: t_39.0_ = 2.15, P = 0.038), but not in 2006 when the data only included first broods (t_39_ = 1.13, P = 0.27; 3y and 4y + males combined).

### Male age and tail length

**Fig. 3:**
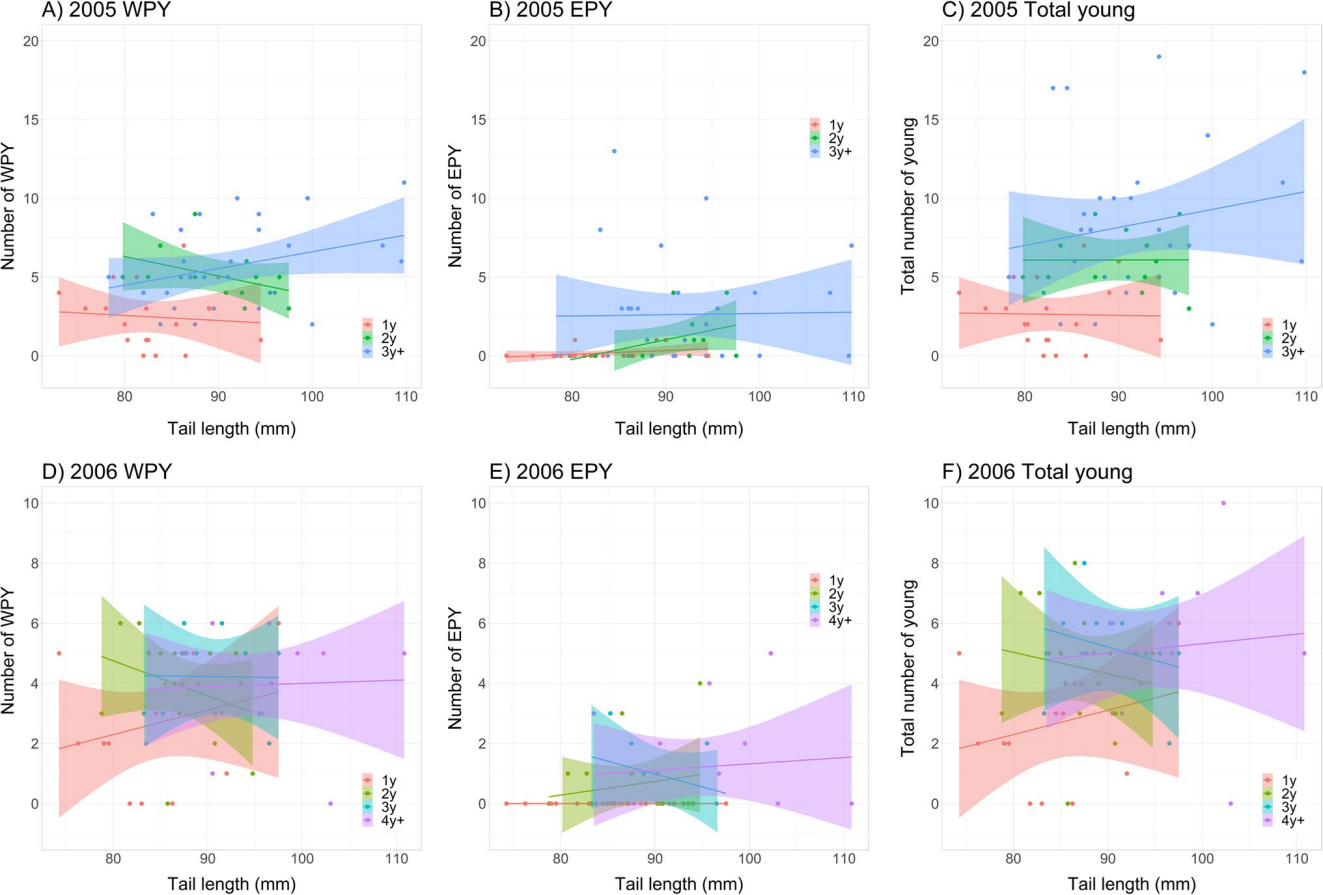
Scatterplots of male fertilization success in barn swallows in the two study years in relation to male age and tail length. Linear regression lines with 95% confidence intervals for each age group are indicated. Test statistics are given in Table 2

If older males have higher fertilization success because they have larger sexual ornaments, as stated by the “sexual selection” hypothesis, we would expect 1) that older males have longer tails, and 2) that tail length predicts fertilization success both across and within age groups. First, we found that male tail length increased incrementally with age, also after the age of 2y (Table 1). However, it is noteworthy that an age effect was also present in females, which suggests that any adaptive value of increased tail length with age should apply to both sexes. A similar age effect was indicated in wing length, though here the contrast was marked only between 1y-olds and older birds (Table 1). Second, we found that male fertilization success increased with tail length, but there were no significant partial effects of tail length in multiple regression models when the effect of male age was accounted for (Fig. 3, Table 2). There was neither any indication of stronger tail length effects in the younger compared to the older age groups (Fig. 3). Thus, there was no support for the prediction of the “sexual selection” hypothesis that male tail length should correlate positively with male fertilization success within age classes. This also held true for the separate analyses of the number of WPY and EPY sired (Fig. 3, Table 2).

**Table 1:** Tail and wing length in relation to age in male and female barn swallows. Age class was based on the year of first breeding (e.g. 1y = 1 year old, 4y +  = 4 years, or older). Values are mean ± SE (N). Tests are linear regression models with age scored as ordinal numbers

		Tail length (mm)		Wing length (mm)	
Year	Age class	Males	Females	Males	Females
2005	1y	83.04 ± 0.66 (50)	75.07 ± 0.65 (42)	121.47 ± 0.32 (50)	120.02 ± 0.38 (42)
	2y	89.97 ± 1.43 (18)	77.02 ± 0.57 (24)	121.78 ± 0.79 (18)	121.40 ± 0.38 (24)
	3y +	91.22 ± 1.28 (34)	79.60 ± 1.01 (23)	122.47 ± 0.47 (34)	120.30 ± 0.57 (23)
	Linear model	F_1,100_ = 39.29, R^2^ = 0.28, P < 0.001	F_1,87_ = 18.78, R^2^ = 0.18, P < 0.001	F_1,100_ = 2.84, R^2^ = 0.03, P = 0.09	F_1,87_ = 0.62, R^2^ = 0.01, P = 0.43
2006	1y	84.66 ± 0.80 (36)	75.69 ± 0.53 (30)	121.58 ± 0.40 (38)	118.73 ± 0.46 (30)
	2y	87.24 ± 1.08 (22)	77.27 ± 1.12 (15)	122.30 ± 0.53 (23)	120.67 ± 0.64 (15)
	3y	92.11 ± 1.84 (10)	78.52 ± 0.93 (13)	121.75 ± 0.85 (10)	121.14 ± 0.56 (14)
	4y +	93.62 ± 1.57 (19)	79.99 ± 1.07 (18)	122.66 ± 0.58 (20)	120.11 ± 0.46 (18)
	Linear model	F_1,85_ = 39.13, R^2^ = 0.32, P < 0.001	F_1,74_ = 16.63, R^2^ = 0.18, P < 0.001	F_1,89_ = 1.96, R^2^ = 0.02, P = 0.17	F_1,75_ = 5.81, R^2^ = 0.07, P = 0.018

**Table 2:** Results of multiple regressions of male fertilization success in male barn swallows with male age and tail length as predictor variables. Age was based on the year of first breeding (e.g. 1y = 1 year old, 4y +  = 4 years or older) and scored as an ordinal number. The data are visualized in Fig. 3

Year	Variable	Number of WPY Estimate ± SE	Number of EPY Estimate ± SE	Total young Estimate ± SE
2005	Intercept	-3.48 ± 3.57	-2.94 ± 3.94	-6.40 ± 5.70
	Age	1.33 ± 0.39**	1.15 ± 0.43**	2.48 ± 0.62***
	Tail length	0.06 ± 0.04	0.02 ± 0.05	0.08 ± 0.07
	Whole model	F_2,57_ = 12.07, R^2^ = 0.30***	F_2,57_ = 5.83, R^2^ = 0.17**	F_2,57_ = 14.69, R^2^ = 0.34***
2006	Intercept	1.84 ± 3.42	-1.20 ± 2.23	0.64 ± 3.92
	Age	0.38 ± 0.26	0.36 ± 0.17*	0.74 ± 0.30*
	Tail length	0.01 ± 0.04	0.01 ± 0.03	0.01 ± 0.05
	Whole model	F_2,51_ = 1.71, R^2^ = 0.06	F_2,51_ = 3.76, R^2^ = 0.13*	F_2,51_ = 5.05, R^2^ = 0.17**

Statistical significance: *P < 0.05, **P < 0.01, *** P < 0.001

The “sexual selection” hypothesis also predicts that females should choose a male for extrapair copulations that have a longer tail than her social mate. In our data over the two study years, we had 58 nests with tail length measurements of both the social male and the extrapair male(s). We found that extrapair males did not have significantly longer tails than the male they cuckolded (paired t-test: t_57_ = 1.73, P = 0.09; using the mean value in cases of multiple extrapair males). In comparison, extrapair males were significantly older than the males they cuckolded (Wilcoxon signed-ranks test: V = 151, N = 60, P < 0.001). Hence, the idea that females use male tail length as a cue to target older males in extrapair mate choice was not supported.

### Male age and fertility

The in vitro analyses of sperm motility revealed that both sperm swimming speed (VCL), the percentage of motile sperm, and the total sperm count increased with male age (Table 3). Total sperm count was affected by the dilution of the collected ejaculate, but we consider it indicative of the original concentration of sperm in the sample. The largest contrasts were observed between males in the two older and the two younger age classes (VCL: t_71_ = 2.79, P = 0.007; percent motile sperm: t_65.9_ = 3.64, P < 0.001; total sperm count: t_71_ = 2.77, P = 0.007). Sperm total length and the length of sperm components did not differ among the age groups (Table 4). Nor did relative midpiece length, which was a significant predictor of fertilization success in tree swallows (Laskemoen et al. 2010). None of the sperm motility or sperm morphology traits correlated significantly with male fertilization success, neither when tested separately, nor when male age was controlled for (tests not shown).

**Table 3:** Sperm behavioural traits in relation to male age in barn swallows. Values are mean ± SE (N). Male age was based on the year of first breeding (e.g. 1y = 1 year old, 4y +  = 4 years, or older)

Age category	Sperm velocity (VCL, µM/s)	Sperm motility (% motile cells)	Total sperm count (number of cells scored)
1y	92.68 ± 1.98 (31)	87.2 ± 1.7 (31)	481.3 ± 55.1 (31)
2y	92.50 ± 2.70 (20)	86.3 ± 1.5 (20)	382.4 ± 55.3 (20)
3y	101.72 ± 4.38 (9)	92.1 ± 1.9 (9)	563.6 ± 95.8 (9)
4y +	100.83 ± 4.01 (13)	92.8 ± 1.2 (13)	708.3 ± 87.6 (13)
Linear regression model	F_1,71_ = 5.87, R^2^ = 0.08, P = 0.018	F_1,71_ = 6.30, R^2^ = 0.08, P = 0.014	F_1,71_ = 5.29, R^2^ = 0.07, P = 0.024

**Table 4:** Sperm morphological traits in relation to male age in barn swallows. Values are mean ± SE (N). Lengths are in µm. Male age was based on the year of first breeding (e.g. 1y = 1 year old, 4y +  = 4 years, or older)

Age category	Total sperm length	Midpiece length	Midpiece:total	Head length
1y	87.65 ± 0.44 (36)	58.72 ± 0.39 (36)	0.670 ± 0.004 (36)	14.05 ± 0.12 (36)
2y	87.43 ± 0.52 (23)	59.24 ± 0.40 (23)	0.678 ± 0.004 (23)	13.98 ± 0.14 (23)
3y	87.44 ± 0.91 (10)	58.29 ± 0.70 (10)	0.667 ± 0.005 (10)	14.26 ± 0.20 (10)
4y +	89.17 ± 0.53 (18)	59.83 ± 0.49 (18)	0.671 ± 0.004 (18)	14.02 ± 0.16 (18)
Linear regression model	F_1,85_ = 3.27, R^2^ = 0.04, P = 0.074	F_1,85_ = 1.86, R^2^ = 0.02, P = 0.18	F_1,85_ = 0.01, R^2^ = 0.00, P = 0.93	F_1,85_ = 0.02, R^2^ = 0.00, P = 0.89

**Fig. 4:**
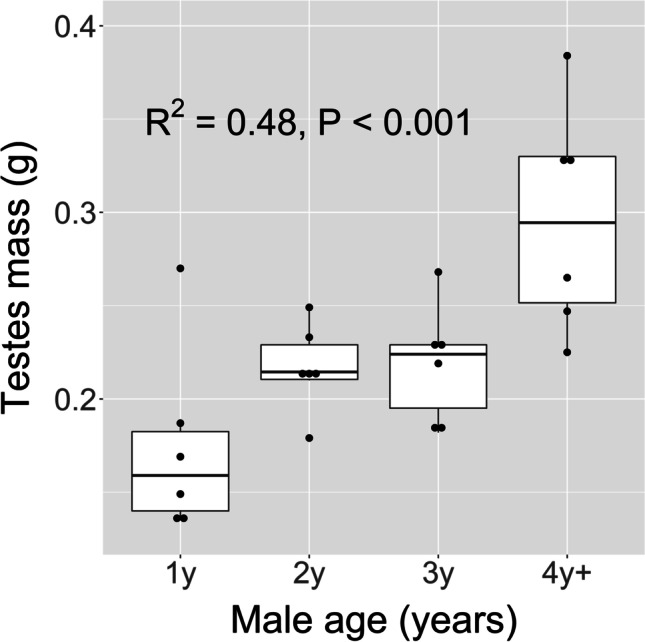
Box plots of testes mass in relation to age in male barn swallows (N = 24). Testes mass is the sum of both testes. Male age is defined from the year of first breeding (e.g. 1y = 1 year old, 4y +  = 4 years, or older). Boxes indicate the interquartile range (IQR), with the central line depicting the median and the whiskers extending to 1.5*IQR, and outliers. R^2^ expresses the proportion of the variance in testes mass explained by male age (scored ordinal numbers) in a linear regression model

Testes mass increased significantly with male age (Fig. 4). The group of 4y + males had almost twice the testes mass of the 1y males. There was no significant partial effect of male tail length in a multiple regression model of the testes mass with both male age and tail length included as predictors (age: t = 3.14, P = 0.005; tail length: t = 1.34, P = 0.20). Male age also explained a significant proportion of the variance in the left (R^2^ = 0.35, P = 0.002) and the right testes (R^2^ = 0.51, P < 0.001) analysed separately. The left testis was larger in 21 of the 24 males. The average mass of the left testis was 0.123 g (SD = 0.035), whereas that of the right testis was 0.104 g (SD = 0.029). This directional asymmetry was statistically significant across males (paired t-test: t_23_ = 4.44, P < 0.001) and follows a common pattern in birds (Calhim and Montgomerie 2015). Testis asymmetry (log [left testis mass] – log [right testis mass]) was however not related to male age (linear regression: R^2^ = 0.03, P = 0.40).

## Discussion

We have documented here that fertilization success increases gradually across age classes in male barn swallows. These results complement our previous study (Lifjeld et al. 2011) where we could only compare two age classes, i.e. 1y and 2y + males. We find that the increase in fertilization success was most pronounced between 1y- and 2y-olds, but also that there was an increase after the second breeding year. In particular, the success in siring EPY was highest among the oldest males (the 3y + group). This result agrees well with a recent study on European barn swallows showing that the probability of siring EPY increased linearly across four male age classes (Michálková et al. 2019). In our previous study, we reported a positive correlation between tail length and fertilization success for the group of 2y + males and discussed whether it might be due to a further age structuring within this composite age group. Here we confirm that both tail length and fertilization success increased after the second breeding year, and that there was no positive relationship between tail length and fertilization success within age groups. Hence, the prediction of the “sexual selection” hypothesis was not supported. We therefore conclude that the age-related increase in male fertilization success is not caused by a female extra-pair preference for long-tailed males in our study population.

Barn swallows may live longer than four years (Saino et al. (2012)) and our age class of 4y + probably contained males that were 5 years or older. Reproductive senescence has been demonstrated in barn swallows for males that are older than 4 years (Møller and de Lope 1999). Likewise, the success in acquiring EPY peaks at the age of four years in wild house sparrows and declines thereafter (Hsu et al. 2017). A similar peak in EPY success was observed in a captive population of house sparrows (Girndt et al. 2018). It is possible that male fertilization success reaches a peak around the age of 4 years with a subsequent decline in reproductive performance also in our population. But unfortunately, we lack the exact age of older males to be able to test for such senescence effects.

If males do not improve their attractiveness through long tail feathers, then why do older males still have longer tails? The alternative explanation to sexual selection is that tail length is a naturally selected trait for optimal flight performance (Norberg 1994; Buchanan and Evans 2000; Rowe et al. 2001). The age-related increase in tail length occurs in both sexes and is possibly an adaptation to faster migration and early arrival on the breeding grounds. Indeed, Matyjasiak (2013) showed that barn swallows with better flight performance arrived earlier on their breeding grounds in Poland. The earlier start of breeding by older individuals with longer tails and wings supports this natural selection explanation. It is also interesting to note that Møller and de Lope (1999) found that barn swallows older than four years attained shorter tails and arrived later in spring, which they interpreted as a senescence effect in migratory performance.

Although females do not seem to use male tail length in mate choice in our population, we cannot exclude a role of female preferences. In theory, females could still prefer older males, assuming that they are able to pick them out by other cues. A preference for male age could be adaptive if older males deliver better genes (Brooks and Kemp 2001). However, theory also holds that older males may deliver poorer genes due to an ageing germline that accumulates deleterious mutations (Lemaître and Gaillard 2017; Monaghan et al. 2020). Not many studies provide empirical evidence on the genetic quality of offspring in relation to paternal age, but a recent study found a negative genetic effect of old paternal age in a halfsib comparison in collared flycatchers *Ficedula albicollis* (Segami et al. 2021). If a similar negative effect applies to barn swallows, then there is no reason to expect any female preference for male age underlying the elevated fertilization success in older males. Still, females might well have an adaptive preference for other traits in extrapair mating, e.g. immune genes (Lindsay et al. 2019; Rekdal et al 2019), in which case males respond adaptively to the potential for increased reproductive success by devoting more resources to extrapair mating effort as they get older.

Our data strongly suggest that older males are more fertile. Both testes size and sperm quality, expressed as sperm swimming speed and motility, increased across the age groups. Testes size is assumed to be a good proxy for sperm production capacity (Laskemoen et al. 2008; Lüpold et al. 2009; Rowe and Pruett-Jones 2011). Several studies have documented that older males have larger testes than young males (Birkhead et al. 1997; Merilä and Sheldon 1999; Graves 2004; Laskemoen et al. 2008), but they have usually revealed a contrast between 1y males and older males only. Here we show that testes size increases also after the second breeding year. The higher fertilization success of 3y + males, especially in the number of EPY sired, may be due to a higher sperm production rate in these males. Higher sperm production allows for more frequent extrapair copulations and/or higher sperm counts per copulation. This will give older males a numerical advantage in sperm competition. An additional effect of higher sperm quality is also possible, since we found a marked difference in sperm swimming speed and motility between the 3y + males and the two younger age groups (Table 3). In sum, older males beyond the second year have improved fertility, which seems a sufficient explanation for their enhanced fertilization success.

We conclude that the age-related increase in male fertilization success in our population of barn swallows reflects a shift in male reproductive effort as predicted by the “life history” hypothesis. This is manifested in higher fertility and an earlier start of breeding in older males. The longer tail feathers in older males are presumably an adaptation to early arrival on the breeding grounds and are thus a naturally selected trait with age-related phenotypic plasticity.

## Supplementary Information

Below is the link to the electronic supplementary material.

Supplementary file 1 (DOCX 19.6 kb)

Supplementary file 2 (XLSX 27.5 kb)

Supplementary file 3 (XLSX 28.5 kb)

Supplementary file 4 (XLSX 15.2 kb)

Supplementary file 5 (XLSX 16.3 kb)

Supplementary file 6 (XLSX 15.6 kb)

Supplementary file 7 (XLSX 12.6 kb)

## Data Availability

The datasets and analytical codes are available as supplementary material.
