# Supplementary material for: When Older Males Sire More Offspring—Increased Attractiveness or Higher Fertility?
Source: Behav Ecol Sociobiol. 2022 Apr 23;76(5):61. doi: 10.1007/s00265-022-03170-0 (PMC9034975; doi:10.1007/s00265-022-03170-0)
Supplement: Supplementary file 1 — (DOCX 19.6 kb) [file 265_2022_3170_MOESM1_ESM.docx]

**Behavioral Ecology and Sociobiology**

**When older males sire more offspring – increased attractiveness or higher fertility?**

Jan T. Lifjeld*, Oddmund Kleven, Frode Fossøy, Frode Jacobsen, Terje Laskemoen, Geir Rudolfsen, Raleigh J. Robertson

* Corresponding author: Natural History Museum, University of Oslo, PO Box 1172 Blindern, NO-0316 Oslo, Norway. Email: [j.t.lifjeld@nhm.uio.no](mailto:j.t.lifjeld@nhm.uio.no).

**Supplementary Information 1**

#R scripts for tables, figures and statistical tests (following the order in the Results chapter)

# Data files should be converted to .txt format before running the scripts

require(ggplot2)

require(tidyverse)

require(plyr)

require(pastecs)

#data files

BS05 = read.table("BS2005.txt", na.strings = "NA",header=TRUE)

fem05=subset(BS05, sex=="female")

male05=subset(BS05, sex=="male")

female=read.table("females2006.txt", na.strings = "NA", header =TRUE)

barnswallow = read.table("BARS2006allmales.txt", na.strings = "NA",header=TRUE)

bsbreeders = read.table("BARS2006onlybreeders.txt", na.strings = "NA",header=TRUE)

bars = read.table("BARStestes.txt", na.strings = "NA",header=TRUE)

pairedWPM_EPM = read.table("WPM_EPM.txt", na.strings = "NA",header=TRUE)

#Figure 1 graphs and tests (Male age and fertilization success)

##remove individuals with unknown age

xx=which(BS05$ageclass != "NA")

BS05=BS05[xx,]

male05=subset(BS05, sex=="male")

##Figure 1A

Fig1A = ggplot(male05, aes(x=ageclass, y=wpy)) + geom_boxplot()+ scale_x_discrete(name="Male age (years)") +

scale_y_continuous(name="Number of WPY sired", limits = c(-1, 20), breaks = c(0,5,10,15,20)) +

theme(text=element_text(size=30)) + ggtitle("A) 2005: WPY")+ geom_dotplot(binaxis='y', stackdir='center', dotsize=0.4) +

annotate("text", x=1.5, y = 17.5, size = 10, parse = TRUE, label = as.character(expression(R^{2}*" = 0.28, P < 0.001")))

Fig1A

test1A = lm(wpy ~age, data = male05)

summary(test1A)

##Figure 1B

Fig1B = ggplot(male05, aes(x=ageclass, y=epy)) + geom_boxplot() + scale_x_discrete(name="Male age (years)") +

scale_y_continuous(name="Number of EPY sired", limits = c(-1, 20), breaks = c(0,5,10,15,20)) +

theme(text=element_text(size=30)) + ggtitle("B) 2005: EPY") + geom_dotplot(binaxis='y', stackdir='center', dotsize=0.4) +

annotate("text", x=1.5, y = 17.5, size = 10, parse = TRUE, label = as.character(expression(R^{2}*" = 0.17, P = 0.001")))

Fig1B

test1B = lm(epy ~age, data = male05)

summary(test1B)

##Figure 1C

Fig1C = ggplot(male05, aes(x=ageclass, y=RS)) + geom_boxplot() + scale_x_discrete(name="Male age (years)") +

scale_y_continuous(name="Total number of young sired", limits = c(-1, 20), breaks = c(0,5,10,15,20)) +

theme(text=element_text(size=30)) + ggtitle("C) 2005: Total young") + geom_dotplot(binaxis='y', stackdir='center', dotsize=0.4) +

annotate("text", x=1.5, y = 17.5, size = 10, parse = TRUE, label = as.character(expression(R^{2}*" = 0.33, P < 0.001")))

Fig1C

test1C = lm(RS ~age, data = male05)

summary(test1C)

##Figure 1D

Fig1D = ggplot(bsbreeders, aes(x=maleage, y=wpy)) + geom_boxplot() + scale_x_discrete(name="Male age (years)") +

scale_y_continuous(name="Number of WPY sired", limits = c(-1, 10), breaks = c(0,2,4,6,8,10)) +

theme(text=element_text(size=30)) + ggtitle("D) 2006: WPY") + geom_dotplot(binaxis='y', stackdir='center', dotsize=0.4) +

annotate("text", x=1.8, y = 8.5, size = 10, parse = TRUE, label = as.character(expression(R^{2}*" = 0.07, P = 0.043")))

Fig1D

test1D = lm(wpy ~magecat, data = bsbreeders)

summary(test1D)

##Figure 1E

Fig1E = ggplot(bsbreeders, aes(x=maleage, y=epysired)) + geom_boxplot() + scale_x_discrete(name="Male age (years)") +

scale_y_continuous(name="Number of EPY sired", limits = c(-1, 10), breaks = c(0,2,4,6,8,10)) +

theme(text=element_text(size=30)) + ggtitle("E) 2006: EPY")+ geom_dotplot(binaxis='y', stackdir='center', dotsize=0.4) +

annotate("text", x=1.8, y = 8.5, size = 10, parse = TRUE, label = as.character(expression(R^{2}*" = 0.14, P = 0.005")))

Fig1E

test1E = lm(epysired ~magecat, data = bsbreeders)

summary(test1E)

##Figure 1F

Fig1F = ggplot(bsbreeders, aes(x=maleage, y=RS)) + geom_boxplot() + scale_x_discrete(name="Male age (years)") +

scale_y_continuous(name="Total number of young sired", limits = c(-1, 10), breaks = c(0,2,4,6,8,10)) +

theme(text=element_text(size=30)) + ggtitle("F) 2006: Total young") + geom_dotplot(binaxis='y', stackdir='center', dotsize=0.4) +

annotate("text", x=1.8, y = 8.5, size = 10, parse = TRUE, label = as.character(expression(R^{2}*" = 0.18, P = 0.001")))

Fig1F

test1F = lm(RS ~magecat, data = bsbreeders)

summary(test1F)

#test of the likelihood of a second brood in relation to male age (2005)

Prob2ndbrood = subset(male05, broods > 0)

xtabs(~broods + age, data=Prob2ndbrood)

chisq.test(Prob2ndbrood$broods, Prob2ndbrood$age, correct=TRUE)

#test of age-related increase in brood size (2006)

broodsize = lm(young ~magecat, data = bsbreeders)

summary(broodsize)

#Figure 2 graphs and tests (Male age and laying date)

##Figure 2A

Fig2A = ggplot(data=subset(male05, !is.na(age)), aes(x=ageclass, y=egglay)) + geom_boxplot() +

theme(text=element_text(size=30)) + geom_dotplot(binaxis='y', stackdir='center', dotsize=0.4) +

scale_x_discrete(name="Male age (years)") + scale_y_continuous(name="Laying date (1 = May 1)") + ggtitle("A) 2005") +

annotate("text", x=2.4, y = 56, size = 10, parse = TRUE, label = as.character(expression(R^{2}*" = 0.37, P < 0.001")))

Fig2A

test2A = lm(egglay ~ age, data = male05)

summary(test2A)

##Figure2B

Fig2B = ggplot(barnswallow, aes(x=maleage, y=datelay1brood)) + geom_boxplot() + theme(text=element_text(size=30)) +

geom_dotplot(binaxis='y', stackdir='center', dotsize=0.4)+ scale_x_discrete(name="Male age (years)") +

scale_y_continuous(name="Laying date (1 = May 1)") + ggtitle("B) 2006") +

annotate("text", x=3.3, y = 37, size = 10, parse = TRUE, label = as.character(expression(R^{2}*" = 0.26, P < 0.001")))

Fig2B

test2B = lm(datelay1brood ~ magecat, data = barnswallow)

summary(test2B)

#Logistic regression of the likelihood of a second brood in relation to male age and laying date

Prob2ndbrood$broods = factor(Prob2ndbrood$broods)

mylogit = glm(broods ~ age + egglay, data = Prob2ndbrood, family = "binomial")

summary(mylogit)

#t-test, number of EPY between 2y and 3y+ males (2005)

epy05=subset(male05, age > "1")

##test of equal variances in EPY

EPYvariance05 = var.test(epy ~ age, data = epy05)

EPYvariance05

##Welch test EPY (unequal variances)

EPYdiff05 = t.test(epy ~ age, data = epy05)

EPYdiff05

#t-test, number of EPY between breeding 2y and 3y+ males (2006)

epy06 = subset(bsbreeders, magecat > "2")

epy06$magecat = ifelse(epy06$magecat==3, 3, ifelse(epy06$magecat==4, 5, ifelse(epy06$magecat==5, 5,3)))

##test of equal variances in EPY

EPYvariance06 = var.test(epysired ~ magecat, data = epy06)

EPYvariance06

##two-group t-test (equal variances)

EPYdiff06 = t.test(epysired ~ magecat, data = epy06, var.equal=TRUE)

EPYdiff06

#t-test, number of EPY between all 2y and 3y+ males (2006)

epy06all = subset(barnswallow, magecat > "2")

epy06all$magecat = ifelse(epy06all$magecat==3, 3, ifelse(epy06all$magecat==4, 5, ifelse(epy06all$magecat==5, 5,3)))

##test of equal variances in EPY

EPYvariance06all = var.test(epysired ~ magecat, data = epy06all)

EPYvariance06all

##two-group t-test (unequal variances)

EPYdiff06all = t.test(epysired ~ magecat, data = epy06all)

EPYdiff06all

#t-test of difference in EPY between 4k and 5k+ males (2006)

epy06older = subset(barnswallow, magecat > "3")

##test of equal variance

EPYvariance06older = var.test(epysired ~ magecat, data = epy06older)

EPYvariance06older

##t-test

EPYdiff06older = t.test(epysired ~ magecat, data = epy06older, var.equal=TRUE)

EPYdiff06older

#t-test of difference total number of young between 2y and 3y+ (2005)

##test of equal variance

RSvar05older = var.test(RS ~ age, data = epy05)

RSvar05older

##t-test

RSdiff05older = t.test(RS ~ age, data = epy05)

RSdiff05older

#t-test of difference total number of young between 2y and 3y+ (2006)

RSoldbreeder = subset(bsbreeders, magecat > "2")

RSoldbreeder$magecat = ifelse(RSoldbreeder$magecat==3, 3, ifelse(RSoldbreeder$magecat==4, 5, ifelse(RSoldbreeder$magecat==5,5,3)))

##test of equal variance

RSvar06older = var.test(RS ~ magecat, data = RSoldbreeder)

RSvar06older

##t-test

RSdiff06older = t.test(RS ~ magecat, data = RSoldbreeder, var.equal=TRUE)

RSdiff06older

#Table 1

##2005

##remove individuals with unknown age

xx=which(BS05$ageclass != "NA")

BS05=BS05[xx,]

fem05=subset(BS05, sex=="female")

male05=subset(BS05, sex=="male")

###tail length by sex

tailmf = ddply(BS05, c("ageclass", "sex"), summarise,

N=sum(!is.na(mean_ts)),

mean = mean(mean_ts, na.rm=TRUE),

sd = sd(mean_ts, na.rm=TRUE),

se = sd / sqrt(N))

tailmf

###test of tail length by age (males)

tail05male = lm(mean_ts ~age, data = male05)

anova(tail05male)

summary(tail05male)

###test of tail length by age (females)

tail05female = lm(mean_ts ~age, data = fem05)

anova(tail05female)

summary(tail05female)

###wing length by sex

wingmf = ddply(BS05, c("ageclass", "sex"), summarise,

N=sum(!is.na(wing)),

mean = mean(wing, na.rm=TRUE),

sd = sd(wing, na.rm=TRUE),

se = sd / sqrt(N))

wingmf

###test of wing length by age (males)

wing05male = lm(wing ~age, data = male05)

anova(wing05male)

summary(wing05male)

###test of wing length by age (females)

wing05female = lm(wing ~age, data = fem05)

anova(wing05female)

summary(wing05female)

##2006

##males

##tail length

maletail = ddply(barnswallow, c("maleage"), summarise,

N=sum(!is.na(mean_ts)),

mean = mean(mean_ts, na.rm=TRUE),

sd = sd(mean_ts, na.rm=TRUE),

se = sd / sqrt(N))

maletail

mtail06 = lm(mean_ts ~ magecat, data = barnswallow)

anova(mtail06)

summary(mtail06)

##wing length

malewing = ddply(barnswallow, c("maleage"), summarise,

N=sum(!is.na(wing)),

mean = mean(wing, na.rm=TRUE),

sd = sd(wing, na.rm=TRUE),

se = sd / sqrt(N))

malewing

mwing06 = lm(wing ~magecat, data = barnswallow)

anova(mwing06)

summary(mwing06)

##females

###tail length females

femaletail06 = ddply(female, c("age"), summarise,

N=sum(!is.na(Mstreamer)),

mean = mean(Mstreamer, na.rm=TRUE),

sd = sd(Mstreamer, na.rm=TRUE),

se = sd / sqrt(N))

femaletail06

ftail06 <- lm(Mstreamer ~ fage,

data = female)

anova(ftail06)

summary(ftail06)

###wing length females

femalewing06 = ddply(female, c("age"), summarise,

N=sum(!is.na(wing)),

mean = mean(wing, na.rm=TRUE),

sd = sd(wing, na.rm=TRUE),

se = sd / sqrt(N))

femalewing06

fwing06 <- lm(wing ~ fage,

data = female)

anova(fwing06)

summary(fwing06)

#Figure 3

Fig3A = ggplot(male05, aes(y=wpy, x=mean_ts, color=ageclass)) +

geom_point(size=3) + theme_light() + geom_smooth(method=lm, aes(fill=ageclass))+

scale_x_continuous(name="Tail length (mm)") + theme(text=element_text(size=30), axis.text.x = element_text(hjust=.5)) +

scale_y_continuous(name="Number of WPY", limits = c(-1, 20), breaks = c(0,5,10,15,20)) +

theme(axis.title.y = element_text(margin = margin(r=10)))+

theme(legend.position = c(0.9, 0.15), legend.title=element_blank()) + labs(title = "A) 2005 WPY")

Fig3A

Fig3B = ggplot(male05, aes(y=epy, x=mean_ts, color=ageclass)) +

geom_point(size=3) + theme_light() + geom_smooth(method=lm, aes(fill=ageclass)) +

scale_x_continuous(name="Tail length (mm)") + theme(text=element_text(size=30), axis.text.x = element_text(hjust=.5)) +

scale_y_continuous(name="Number of EPY", limits = c(-1, 20), breaks = c(0,5,10,15,20)) +

theme(axis.title.y = element_text(margin = margin(r=10))) +

theme(legend.position = c(0.9, 0.7), legend.title=element_blank()) + labs(title = "B) 2005 EPY")

Fig3B

Fig3C = ggplot(male05, aes(y=RS, x=mean_ts, color=ageclass)) +

geom_point(size=3) + theme_light() + geom_smooth(method=lm, aes(fill=ageclass)) + scale_x_continuous(name="Tail length (mm)") +

theme(text=element_text(size=30), axis.text.x = element_text(hjust=.5)) +

scale_y_continuous(name="Total number of young", limits = c(-1, 20), breaks = c(0,5,10,15,20)) +

theme(axis.title.y = element_text(margin = margin(r=10))) +

theme(legend.position = c(0.9, 0.15), legend.title=element_blank()) + labs(title = "C) 2005 Total young")

Fig3C

Fig3D = ggplot(bsbreeders, aes(y=wpy, x=mean_ts, color=maleage)) +

geom_point(size=3) + theme_light() + geom_smooth(method=lm, aes(fill=maleage)) + scale_x_continuous(name="Tail length (mm)") +

theme(text=element_text(size=30), axis.text.x = element_text(hjust=.5)) +

scale_y_continuous(name="Number of WPY", limits = c(-1, 10), breaks = c(0,2,4,6,8,10)) +

theme(axis.title.y = element_text(margin = margin(r=10)))+

theme(legend.position = c(0.9, 0.15), legend.title=element_blank()) + labs(title = "D) 2006 WPY")

Fig3D

Fig3E = ggplot(bsbreeders, aes(y=epysired, x=mean_ts, color=maleage)) +

geom_point(size=3) + theme_light() + geom_smooth(method=lm, aes(fill=maleage)) + scale_x_continuous(name="Tail length (mm)") +

theme(text=element_text(size=30), axis.text.x = element_text(hjust=.5)) +

scale_y_continuous(name="Number of EPY", limits = c(-1, 10), breaks = c(0,2,4,6,8,10)) +

theme(axis.title.y = element_text(margin = margin(r=10))) +

theme(legend.position = c(0.9, 0.7), legend.title=element_blank()) + labs(title = "E) 2006 EPY")

Fig3E

Fig3F = ggplot(bsbreeders, aes(y=RS, x=mean_ts, color=maleage)) +

geom_point(size=3) + theme_light() + geom_smooth(method=lm, aes(fill=maleage)) + scale_x_continuous(name="Tail length (mm)") +

theme(text=element_text(size=30), axis.text.x = element_text(hjust=.5)) +

scale_y_continuous(name="Total number of young", limits = c(-1, 10), breaks = c(0,2,4,6,8,10)) +

theme(axis.title.y = element_text(margin = margin(r=10))) +

theme(legend.position = c(0.9, 0.15), legend.title=element_blank()) + labs(title = "F) 2006 Total young")

Fig3F

#Table 2

##2005 WPY

###test with interaction

WPYint05 = lm(wpy ~age + mean_ts + age:mean_ts, data = male05)

summary(WPYint05)

###test without interaction

WPYmreg05 = lm(wpy ~age + mean_ts, data = male05)

summary(WPYmreg05)

##2005 EPY

###test with interaction

EPYint05 = lm(epy ~age + mean_ts + age:mean_ts, data = male05)

summary(EPYint05)

###test without interaction

EPYmreg05 = lm(epy ~age + mean_ts, data = male05)

summary(EPYmreg05)

##2005 WPY + EPY

###test with interaction

RSint05 = lm(RS ~age + mean_ts + age:mean_ts, data = male05)

summary(RSint05)

###test without interaction

RSmreg05 = lm(RS ~age + mean_ts, data = male05)

summary(RSmreg05)

##2006 WPY

###test with interaction

WPYint06 = lm(wpy ~magecat + mean_ts + magecat:mean_ts, data = bsbreeders)

summary(WPYint06)

###test without interaction

WPYmreg06 = lm(wpy ~magecat+ mean_ts, data = bsbreeders)

summary(WPYmreg06)

##2006 EPY

###test with interaction

EPYint06 = lm(epysired ~magecat+ mean_ts + magecat:mean_ts, data = bsbreeders)

summary(EPYint06)

###test without interaction

EPYmreg06 = lm(epysired ~magecat+ mean_ts, data = bsbreeders)

summary(EPYmreg06)

##2006 WPY + EPY

###test with interaction

RSint06 = lm(RS ~magecat+ mean_ts + magecat:mean_ts, data = bsbreeders)

summary(RSint06)

###test without interaction

RSmreg06 = lm(RS ~magecat+ mean_ts, data = bsbreeders)

summary(RSmreg06)

#Paired t-test tail length WP male versus EP male

pairedWPM_EPM.long = pairedWPM_EPM%>%

gather(key="group", value ="Tail", tailWPM, tailEPM)

pairedttest_tail = t.test(Tail ~ group, data = pairedWPM_EPM.long, paired = TRUE)

pairedttest_tail

#Wilcoxon test age WP male versus EP male

wilcox.test(pairedWPM_EPM$AgeWPM, pairedWPM_EPM$AgeEPM, paired = TRUE)

#Table 3

##VCL by age

VCL = ddply(barnswallow, c("maleage"), summarise,

N=sum(!is.na(wVCL)),

mean = mean(wVCL, na.rm=TRUE),

sd = sd(wVCL, na.rm=TRUE),

se = sd / sqrt(N))

VCL

test.VCL = lm(wVCL ~ magecat, data = barnswallow)

summary(test.VCL)

##Prop motile cells by age

motile = ddply(barnswallow, c("maleage"), summarise,

N=sum(!is.na(wMotilePct)),

mean = mean(wMotilePct, na.rm=TRUE),

sd = sd(wMotilePct, na.rm=TRUE),

se = sd / sqrt(N))

motile

test.motile = lm(wMotilePct ~ magecat, data = barnswallow)

summary(test.motile)

##Total sperm count by age

TotalCount = ddply(barnswallow, c("maleage"), summarise,

N=sum(!is.na(Sum_TotalCount)),

mean = mean(Sum_TotalCount, na.rm=TRUE),

sd = sd(Sum_TotalCount, na.rm=TRUE),

se = sd / sqrt(N))

TotalCount

test.TotalCount = lm(Sum_TotalCount ~ magecat, data = barnswallow)

summary(test.TotalCount)

#Sperm motility differences between two youmger and two older age classes

barnswallow$magecat = ifelse(barnswallow$magecat==2, 2, ifelse(barnswallow$magecat==3, 2,

ifelse(barnswallow$magecat==4, 4, ifelse(barnswallow$magecat==5, 4,0))))

##test of equal variance VCL

var.VCL = var.test(wVCL ~ magecat, data = barnswallow)

var.VCL

##t-test VCL

VCL2 = t.test(wVCL ~ magecat, data = barnswallow, var.equal=TRUE)

VCL2

##test of equal variance Prop motile cells

var.motile = var.test(wMotilePct ~ magecat, data = barnswallow)

var.motile

##t-test motile (unequal variances)

motile2 = t.test(wMotilePct ~ magecat, data = barnswallow)

motile2

##test of equal variance Total Sperm Count

var.TotalCount = var.test(Sum_TotalCount ~ magecat, data = barnswallow)

var.TotalCount

##t-test TotalCount

TotalCount2 = t.test(Sum_TotalCount ~ magecat, data = barnswallow, var.equal=TRUE)

TotalCount2

#Table 4

##Total sperm length

barnswallow = read.table("BARS2006allmales.txt", na.strings = "NA",header=TRUE)

TSL = ddply(barnswallow, c("maleage"), summarise,

N=sum(!is.na(total)),

mean = mean(total, na.rm=TRUE),

sd = sd(total, na.rm=TRUE),

se = sd / sqrt(N))

TSL

test.TSL = lm(total ~magecat, data = barnswallow)

summary(test.TSL)

##Midpiece length

midpiece = ddply(barnswallow, c("maleage"), summarise,

N=sum(!is.na(midpiece)),

mean = mean(midpiece, na.rm=TRUE),

sd = sd(midpiece, na.rm=TRUE),

se = sd / sqrt(N))

midpiece

test.midpiece = lm(midpiece ~magecat, data = barnswallow)

summary(test.midpiece)

##Midpiece length:TSL

midpiece.total = ddply(barnswallow, c("maleage"), summarise,

N=sum(!is.na(mp.total)),

mean = mean(mp.total, na.rm=TRUE),

sd = sd(mp.total, na.rm=TRUE),

se = sd / sqrt(N))

midpiece.total

test.midpiece.total = lm(mp.total ~magecat, data = barnswallow)

summary(test.midpiece.total)

#Head length

head = ddply(barnswallow, c("maleage"), summarise,

N=sum(!is.na(head)),

mean = mean(head, na.rm=TRUE),

sd = sd(head, na.rm=TRUE),

se = sd / sqrt(N))

head

test.head = lm(head ~magecat, data = barnswallow)

summary(test.head)

#Figure 4

bars$log_RTM = log(bars$R.testismass)

bars$log_LTM = log(bars$L.testismass)

bars$FAtestes = bars$log_LTM - bars$log_RTM

##Fig4 graph

Fig4 = ggplot(bars, aes(x=age, y=Combtestesmass)) + geom_boxplot() +

theme(text=element_text(size=30)) + geom_dotplot(binaxis='y', stackdir='center', dotsize=0.4) +

scale_x_discrete(name="Male age (years)") + scale_y_continuous(name="Testes mass (g)", limits = c(0.13, 0.40)) +

annotate("text", x=2, y = 0.35, size = 10, parse = TRUE, label = as.character(expression(R^{2}*" = 0.48, P < 0.001")))

Fig4

##mult regr of testes mass by age, tail and the interaction

testes.interaction = lm(Combtestesmass ~ agecat + mean_ts + agecat:mean_ts, data = bars)

summary(testes.interaction)

##mult regr of testes mass by age and tail (interaction removed)

testes.nointeraction = lm(Combtestesmass ~ agecat + mean_ts, data = bars)

summary(testes.nointeraction)

##left testis by age

left.testis = lm(L.testismass ~agecat, data = bars)

summary(left.testis)

##right testis by age

right.testis = lm(R.testismass ~agecat, data = bars)

summary(right.testis)

#left testis descriptive statistics

stat.desc(bars$L.testismass)

#right testis descriptive statistics

stat.desc(bars$R.testismass)

#test of directional asymmetry in testes

bars.long = bars%>%

gather(key="group", value="Testismass", L.testismass, R.testismass)

paired.ttest.testes= t.test(Testismass ~ group, data = bars.long, paired = TRUE)

paired.ttest.testes

#test of directional testis asymmetry by age

FA = lm(FAtestes ~agecat, data = bars)
